# Exclusion of pathogenic promoter region variants and identification of novel nonsense mutations in the zinc finger E-box binding homeobox 1 gene in posterior polymorphous corneal dystrophy

**Published:** 2013-03-15

**Authors:** Pejman Bakhtiari, Ricardo F. Frausto, Ashley N. Roldan, Cynthia Wang, Fei Yu, Anthony J. Aldave

**Affiliations:** The Jules Stein Eye Institute, David Geffen School of Medicine at UCLA, Los Angeles, CA

## Abstract

**Purpose:**

To report the identification of five novel nonsense mutations in the zinc finger E-box binding homeobox 1 (*ZEB1*) gene and exclusion of promoter region mutations in individuals without *ZEB1* coding region mutations in posterior polymorphous corneal dystrophy (PPCD).

**Methods:**

Slit-lamp examination and DNA collection were performed for individuals diagnosed with PPCD and, when available, affected and unaffected family members. Genomic DNA prepared from peripheral blood leukocytes and buccal epithelial cells underwent PCR amplification and automated sequencing of the *ZEB1* gene and 1 kb 5′ of *ZEB1*, presumably containing the *ZEB1* promoter region.

**Results:**

Thirteen unrelated individuals with PPCD were identified, and genomic DNA was collected from each individual. *ZEB1* mutations were identified in six of the 13 probands, five of which were novel: p.(Gly150Alafs*36; spontaneous), p.(His230Argfs*7), p.(Ser638Cysfs*5), p.(Glu1039Glyfs*6), and p.(Gln884Argfs*37). Screening of the *ZEB1* promoter region in 31 probands with PPCD without a *ZEB1* coding region mutation identified only two known single nucleotide polymorphisms (SNPs) whose frequency in the affected probands did not differ significantly from that in the general population.

**Conclusions:**

We report five novel frame-shift mutations, one confirmed as spontaneous, in the *ZEB1* gene associated with PPCD, bringing the total number of reported pathogenic mutations to 24, and the percentage of PPCD associated with *ZEB1* mutations to 32%. The absence of *ZEB1* promoter region mutations in probands without a *ZEB1* coding region mutation indicates that other genetic loci, such as the PPCD1 locus, are involved in the pathogenesis of PPCD.

## Introduction

Posterior polymorphous corneal dystrophy (PPCD; MIM 122000) is an autosomal dominant corneal dystrophy associated with characteristic morphologic endothelial abnormalities and, in severe cases, endothelial decompensation. Although an uncommon inherited disorder, PPCD has been associated with a number of other ocular disorders, including primary open angle and secondary angle-closure glaucoma [1,2], as well as non-keratoconic corneal steepening [3,4] and keratoconus [5,6]. A number of associated extraocular manifestations, including abdominal hernia and hydrocele formation, distinguish PPCD from the majority of the other corneal dystrophies, which are traditionally considered isolated corneal disorders [7,8]. PPCD also differs from the majority of other corneal dystrophies in that locus heterogeneity has been reported, with linkage reported to chromosomes 10 (the PPCD3 locus) [9] and 20 (the PPCD1 locus) [1]. Krafchak and colleagues reported frameshift mutations in the zinc finger E-box binding homeodomain 1 gene (*ZEB1* gene; OMIM 189909) in the PPCD3 locus in five of 11 probands and demonstrated altered endothelial expression of the collagen IV, alpha 3 (*COL4A3*; OMIM 120070) gene in the corneal endothelium of an affected individual, leading to their proposed theory of pathogenesis of PPCD3 [8]. We confirmed the role of *ZEB1* in PPCD3 by reporting eight additional frameshift mutations in 32 probands who were screened, and provided additional evidence to support the role of *ZEB1* in negative regulation of *COL4A3* transcription [7].

To date, 19 coding region mutations, all nonsense, have been identified in the *ZEB1* gene in 65 families with PPCD [7,8,10-12]. We report the identification of six *ZEB1* coding region mutations in 13 additional probands, five of which are novel and one that was confirmed to be spontaneous. We also report the absence of pathogenic sequence variants in the *ZEB1* promoter region in 31 probands without *ZEB1* coding region mutations. Our results indicate that truncating *ZEB1* mutations are present in approximately one third of probands with PPCD, with a unique mutation identified in every proband except one screened to date.

## Methods

The authors followed the tenets of the Declaration of Helsinki in the treatment of the subjects. Study approval was obtained from the Institutional Review Board at the University of California, Los Angeles (UCLA IRB # 94–07–243-(14–33A), 02–10–092-(4,11), 10–001932).

### Patient identification/deoxyribonucleic acid collection and preparation

Patients examined on the Cornea Service at the Jules Stein Eye Institute (by Dr. Anthony Aldave) or at the Kansas University Eye Center (by Dr. John Sutphin) were diagnosed with PPCD based on the presence of characteristic corneal endothelial changes in one or both eyes: an endothelial band with parallel borders typically associated with white flaky-appearing material along the edge of the band; single or grouped vesicular endothelial changes, typically associated with a surrounding gray halo; and either discreet or confluent geographic gray endothelial opacities. After individuals were offered enrollment in the study by Dr. Anthony Aldave, and after informed consent was obtained, a peripheral blood sample, a buccal epithelial sample (Cyto-Soft Cytology Brush; Medical Packaging Corporation, Camarillo, CA), or a saliva sample (Oragene saliva collection kits; DNA Genotek, Inc., Kanata, Canada) was collected as a source of genomic DNA. Unrelated, unaffected, healthy volunteers were recruited to serve as controls. Genomic DNA was prepared from the peripheral blood leukocytes and buccal epithelial cells using the FlexiGene DNA and QIAamp DNA Blood Mini Kits (Qiagen, Valencia, CA), respectively.

### Polymerase chain reaction amplification

Each of the nine exons of *ZEB1* was amplified using primers and conditions previously described by Krafchak et al. with the exception of exon 1, which was amplified using a custom-designed oligonucleotide pair described previously [7,8]. An alternative exon 1 and the 1 Kb upstream of the initiation methionine (ATG), containing the core and putative distal promoter regions were amplified using custom-designed oligonucleotide pairs (Table 1). Each 25 μl reaction contained 50 mM Tris-HCl (pH 9.0, 25 °C), 20 mM NH_4_Cl, 2.5 mM MgSO_4_, 200 mM each deoxynucleotide triphosphate (dNTP) plus 20 mM 7-deaza-2´-deoxyguanosine 5´-triphosphate, 0.5 M Betaine, 2.5 μl dimethyl sulfoxide, 150 mM Trehalose, 0.002% Tween-20, 0.12 mM of each primer, 0.5 units of RedTaq Genomic DNA Polymerase (Sigma-Aldrich, St. Louis, MO), and approximately 20 ng of genomic DNA. Thermal cycling was performed in an iCycler Thermal Cycler (Bio-Rad, Hercules, CA).

**Table 1:** Primers used for screening alternative exon 1 and the 1 Kb upstream of the zinc finger E-box binding homeobox 1 (*ZEB1*) gene.

***ZEB1***	**Forward primer 5’-3’**	**Reverse primer 5’-3’**	**Annealing Temp**
Alternative exon 1	GTGGAGAGATGACTTGTTATAGCA	GTGGTTCAGACTCACAGTC	54 °C
Promoter: Proximal Region	GCCGATGCTTCTTGCCTTAAG	GCTGTCGGAGTTGGAAAGGTAAAG	56 °C
Promoter: Distal Region	CCAGACCGCGATCCCTTCCTTG	CTCCGCCACTCACCGTATTG	56 °C

### Deoxyribonucleic acid sequencing

Purification of the PCR products was achieved by incubating 15–30 ng of amplified DNA with 5 U Exonuclease I and 0.5 U shrimp alkaline phosphatase (USB Corp., Cleveland, OH) for 15 min at 37 °C. After inactivation of the nucleases for 15 min at 80 °C, sequencing reactions were performed by adding 2 µl of Big Dye Terminator Mix v3.1 (Applied Biosystems, Foster City, CA), 2 µl of SeqSaver (Sigma-Aldrich), and 0.2 µl of primer (10 pmoles/µl). Samples were denatured at 96 °C for 2 min and then cycled 25 times at 96 °C for 10 s, 50 °C for 5 s, and 60 °C for 4 min. Unincorporated nucleotides were removed using the CleanSeq reagent and an SPRI plate (Agencourt Bioscience Corporation, Beverly, MA) following the manufacturer’s instructions and then analyzed on an ABI-3100 Genetic Analyzer (Applied Biosystems) after resuspension in 0.1 mM EDTA. Coding region nucleotide sequences (including the donor and acceptor splice sites) were read manually by comparison to the *ZEB1* cDNA sequences (GenBank accession number NM_030751 and NM_001128128.2), while promoter region sequences were compared to the *ZEB1* RefSeqGene sequence (GenBank accession number NG_017048.1). The description of the identified sequence variants adhered to the Human Genome Variation Society (HGVS) nomenclature guidelines.

### Statistical analyses

The Fisher exact test was used to identify an association between *ZEB1* promoter region sequence variants and PPCD in probands without a *ZEB1* coding region mutation. The binomial proportion test was used to compare the percentage of all probands with PPCD demonstrating a *ZEB1* promoter region sequence variant with the prevalence in the general population as reported in HapMap.

## Results

### Patient Identification and screening of the zinc finger E-box binding homeobox 1 gene coding region

**Figure 1:**
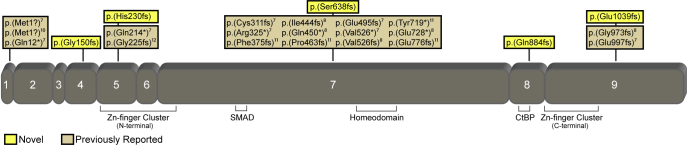
Depiction of the zinc finger E-box binding homeobox 1 (ZEB1) protein, demonstrating the location of the five novel mutations and 19 previously reported mutations. The mutation nomenclature is presented according the Human Genome Variation Society (HGSV) guidelines, and thus may be different from the nomenclature used in original publications. Important functional domains are also depicted.

Thirteen probands diagnosed with PPCD based on the presence of characteristic clinical features were enrolled into the study and provided DNA for genetic analysis. Ten of the 13 probands were female, and the average age at the time of enrollment was 36 years of age (range, 5-77 years). Screening of the *ZEB1* coding region in the 13 PPCD probands identified six nonsense mutations in the heterozygous state, of which five are novel: c.449delG (p.(Gly150Alafs*36)), c.689_690delAT (p.(His230Argfs*7)), c.1913_1914delCA (p.(Ser638Cysfs*5)), c.2650delC (p.(Gln884Argfs*37)), and c.3116_3117delAG (p.(Glu1039Glyfs*6)). The sixth mutation identified, c.1576dupG (p.(Val526Glyfs*3)), has been previously reported [8]. Located in exons 4 and 5, the p.(Gly150Alafs*36) and p.(His230Argfs*7) mutations, respectively, are predicted to result in the loss or disruption of multiple important functional domains of the ZEB1 protein, including the C-terminal binding protein and SMAD binding domains, the homeodomain, and the N- and C-terminal zinc-finger clusters (Figure 1). The p.(Ser638Cysfs*5) mutation located in exon 7 is predicted to result in the loss of the CtBP binding domains, the homeodomain, and the C-terminal zinc-finger cluster. Located in exon 8, the p.(Gln884Argfs*37) mutation is predicted to result in the loss of the C-terminal zinc-finger cluster. The p.(Glu1039Glyfs*6) mutation located in exon 9 is not predicted to result in the loss of any of these important functional domains.

**Figure 2:**
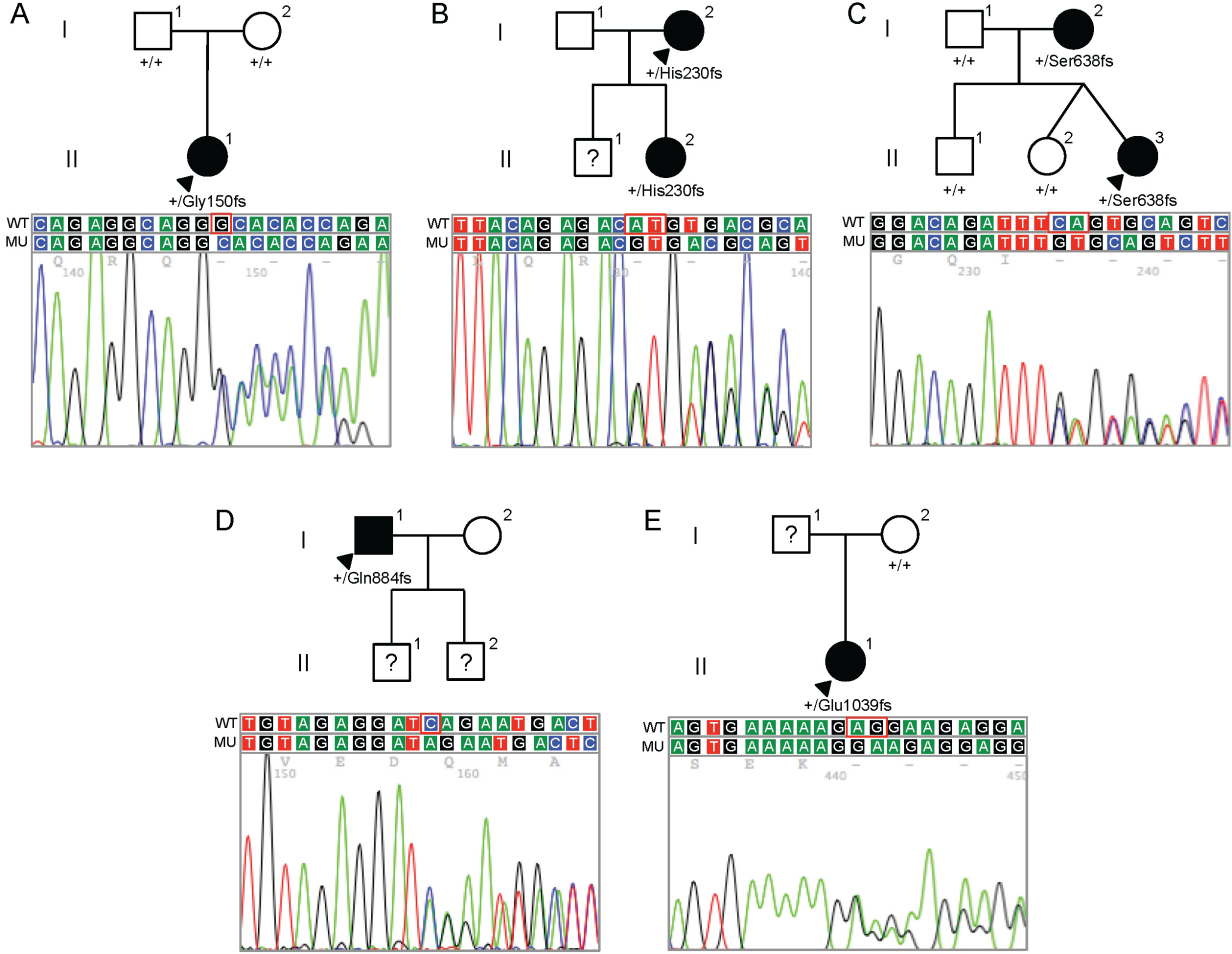
Pedigrees and zinc finger E-box binding homeobox 1 (*ZEB1*) sequences for the five families in which novel zinc finger E-box binding homeobox 1 mutations were identified. In each pedigree, the presence of the wild-type allele (designated by the + symbol) or the mutant allele is indicated below the symbol of each individual in whom DNA collection and zinc finger E-box binding homeobox 1 (*ZEB1*) gene screening were performed. Filled symbols represent affected individuals, open symbols represent unaffected individuals, and question marks indicate individuals of undetermined affected status. Arrowheads indicate probands. Beneath each pedigree, chromatograms demonstrating the identified mutation (MU) and the wild-type DNA sequence (WT) are shown.

**Figure 3:**
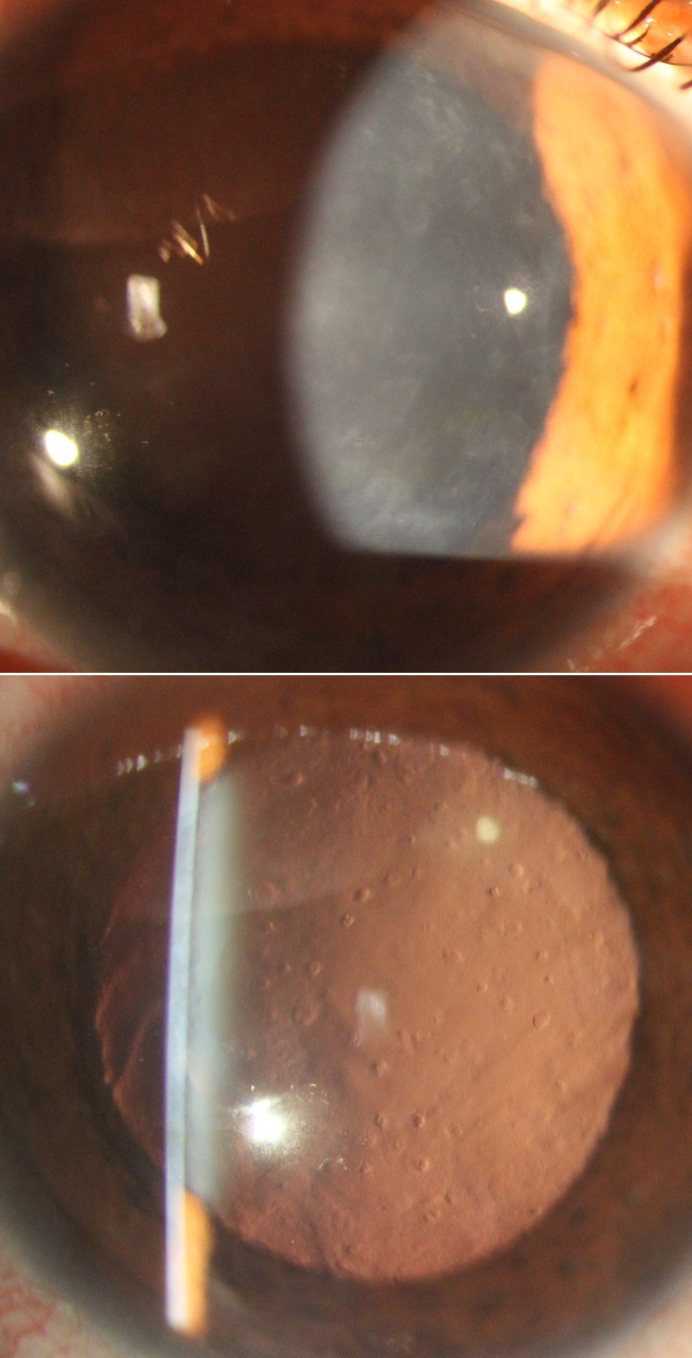
Slit-lamp photomicrographs of a patient with posterior polymorphous corneal dystrophy 3 (PPCD3) secondary to p.(Gln884Argfs*37) mutation in the zinc finger E-box binding homeobox 1 gene (*ZEB1*). Diffusely distributed geographic gray-white Descemet membrane opacities are seen on direct illumination (**A**) that appear as round and oval-shaped “vesicles” on retroillumination, consistent with posterior polymorphous corneal dystrophy (PPCD).

Screening of available affected and unaffected family members of each affected proband was performed (Figure 2). Both parents of the proband in whom the p.(Gly150Alafs*36) mutation was identified were clinically unaffected, and neither demonstrated the mutation identified in the proband. Paternity testing confirmed that the father was the biologic father, indicating that the mutation present in the proband most likely arose spontaneously. The only available parent of the proband demonstrating the p.(Glu1039Glyfs*6) mutation was unaffected and did not demonstrate the mutation identified in the heterozygous state in the proband. In contrast, the p.(His230Argfs*7) and p.(Ser638Cysfs*5) mutations were identified in an affected parent and child in the heterozygous state. The p.(Gln884Argfs*37) mutation was identified in the heterozygous state in an affected individual, although no other family members were available for analysis (Figure 3).

### Screening of the zinc finger E-box binding homeobox 1 gene promoter region

Thirty-one probands with PPCD in whom *ZEB1* coding region mutations were not identified were selected for screening of the putative *ZEB1* promoter located within a 1 kb region upstream of the ATG start codon. Two known single nucleotide polymorphisms, −933T>G (rs3737179) and −803G>C (rs3737180), were identified together in the heterozygous state in six of the 31 (19.3%) probands (the minor allele haplotype). To test for an association between the minor allele haplotype and the absence of *ZEB1* coding sequence variants, we compared the prevalence of this haplotype in the general population to that in 14 probands with PPCD3 (in whom a *ZEB1*coding region mutation had been identified). None of the probands with PPCD3 demonstrated the minor allele of either single nucleotide polymorphism, although the 0% prevalence was not statistically significantly less than the 12.4% prevalence of the haplotype in the general population (data from HapMap; binomial proportion test p=0.19) or the 19.3% prevalence in probands with PPCD in whom *ZEB1* coding region mutations were not identified (Fisher exact test p=0.16). Additionally, the 13.3% (6/45) prevalence of this haplotype in all probands with PPCD (ZEB1 and non-ZEB1) was not significantly different from that in the general population (binomial proportion test p=0.85).

## Discussion

With the current report of five novel *ZEB1* mutations in 13 probands with PPCD, the total number of reported mutations identified in *ZEB1* associated with PPCD increases to 24, and the percentage of probands with PPCD in whom a *ZEB1* coding region mutation has been identified increases to 32% (25/78) [7,8,10-12]. As no pathogenic sequence variants were identified in the putative promoter region in the 31 probands with PPCD without a *ZEB1* coding region mutation, we can conclude that other genetic loci, such as the PPCD1 locus on chromosome 20, are involved in the pathogenesis of PPCD.

We report the first *ZEB1* mutation that is not unique to the family in which it was identified, p.(Val526Glyfs*3), which was originally reported by Krafchak and colleagues [8]. The Caucasian pedigree in whom we identified this mutation could be related to the Caucasian family reported by Krafchak and colleagues. However, as the mutation was not identified in either parent of the proband that we report, it may also represent a spontaneous mutation (although this could not be confirmed as we did not perform paternity testing). Two different spontaneous mutations in *ZEB1* have been reported by Krafchak and colleagues, and thus, the novel p.(Gly150Alafs*36) mutation that we report represents the third reported spontaneous mutation [8]. Although spontaneous mutations are therefore implicated in 12% (3/25) of probands with PPCD with *ZEB1* mutations, these mutations have been identified in only one other gene associated with a corneal dystrophy, the transforming growth factor, beta-induced (*TGFBI*) gene [13,14]. Given this, as well as the fact that the majority of the corneal dystrophies are dominantly inherited and associated with complete penetrance, clinicians commonly rely on the presence of a positive family history in diagnosing corneal dystrophies. Therefore, identifying and reporting spontaneous pathogenic mutations associated with corneal dystrophies is important to ensure that clinicians consider the possibility of a dominantly inherited corneal dystrophy even in the absence of a family history.

Four of the five novel mutations that we report are predicted to result in the loss of one or more domains critical to the function of the ZEB1 protein. The ZEB1 protein is generated from nine coding exons that contain two zinc finger clusters (N-terminal and C-terminal), a homeodomain, and SMAD and CtBP binding domains (Figure 1). The zinc finger domains are DNA-binding motifs, while the homeodomain has been reported to interact with the N-terminal zinc finger cluster, in an intraprotein interaction [15]. SMAD proteins are the transducers of TGF-β signaling, relaying signals from cell-surface receptors to the nucleus, where the proteins activate transcription of specific target genes [16]. Thus, the loss of any one of these domains could significantly alter the function of the truncated protein product. As the fifth novel mutation that we describe is located downstream of the C-terminal zinc finger cluster in exon 9, haploinsufficiency likely occurs primarily as a consequence of nonsense-mediated messenger ribonucleic acid decay (NMD) [17,18]. Alternatively, putative functional properties of the deleted portion of the p.(Glu1039Glyfs*6) mutant protein may be crucial to protein function (e.g., tertiary folding, intracellular localization, or signal transduction), leading to a non-functional protein and subsequently to PPCD. To determine the effects of the novel *ZEB1* mutations that we report, we are currently performing a series of experiments to detect the subcellular localization of the corresponding mutant ZEB1 protein in transfected primary human corneal endothelial cells.
